# Global–local multi-stage temporal convolutional network for cataract surgery phase recognition

**DOI:** 10.1186/s12938-022-01048-w

**Published:** 2022-11-30

**Authors:** Lixin Fang, Lei Mou, Yuanyuan Gu, Yan Hu, Bang Chen, Xu Chen, Yang Wang, Jiang Liu, Yitian Zhao

**Affiliations:** 1 College of Mechanical Engineering, Zhejiang University of Technology, Hangzhou, 310014 China; 2 Cixi Institute of Biomedical Engineering, Ningbo Institute of Materials Technology and Engineering, Chinese Academy of Sciences, Ningbo, China; 3 Department of Computer Science and Engineering, Southern University of Science and Technology, Shenzhen, 518055 China; 4 Department of Ophthalmology, Shanghai Aier Eye Hospital, Shanghai, China; 5 Department of Ophthalmology, Shanghai Aier Qingliang Eye Hospital, Shanghai, China; 6 Aier Eye Hospital, Jinan University, No. 601, Huangpu Road West, Guangzhou, China; 7 Aier School of Ophthalmology, Central South University Changsha, Changsha, Hunan China; 8 Zhejiang Engineering Research Center for Biomedical Materials, Cixi Institute of Biomedical Engineering, Ningbo Institute of Materials Technology and Engineering, Chinese Academy of Sciences, Ningbo, 315300 China; 9 Aerospace Information Research Institute, Chinese Academy of Sciences, Beijing, China

**Keywords:** Surgical phase recognition, Temporal convolutional networks, Cataract surgery videos, Deep learning

## Abstract

**Background:**

Surgical video phase recognition is an essential technique in computer-assisted surgical systems for monitoring surgical procedures, which can assist surgeons in standardizing procedures and enhancing postsurgical assessment and indexing. However, the high similarity between the phases and temporal variations of cataract videos still poses the greatest challenge for video phase recognition.

**Methods:**

In this paper, we introduce a global–local multi-stage temporal convolutional network (GL-MSTCN) to explore the subtle differences between high similarity surgical phases and mitigate the temporal variations of surgical videos. The presented work consists of a triple-stream network (i.e., pupil stream, instrument stream, and video frame stream) and a multi-stage temporal convolutional network. The triple-stream network first detects the pupil and surgical instruments regions in the frame separately and then obtains the fine-grained semantic features of the video frames. The proposed multi-stage temporal convolutional network improves the surgical phase recognition performance by capturing longer time series features through dilated convolutional layers with varying receptive fields.

**Results:**

Our method is thoroughly validated on the CSVideo dataset with 32 cataract surgery videos and the public Cataract101 dataset with 101 cataract surgery videos, outperforming state-of-the-art approaches with 95.8% and 96.5% accuracy, respectively.

**Conclusions:**

The experimental results show that the use of global and local feature information can effectively enhance the model to explore fine-grained features and mitigate temporal and spatial variations, thus improving the surgical phase recognition performance of the proposed GL-MSTCN.

## Introduction

Computer-assisted surgery (CAS) systems play a crucial role in the development of modern surgery, which can prevent improper decisions resulting from complex surgical procedures, thereby reducing the risk of postoperative complications, irreversible injuries, and unnecessary pain [1]. A key task required of CAS systems is the recognition of the surgical phase, as any form of assistance that is not manually triggered and directed by the surgical team requires an understanding of the surgical environment, the human interactions that occur in the room, and their evolution near the patient and elsewhere [2]. By automatically recognizing and evaluating current surgical scenarios, CAS systems can provide intraoperative decision support, improve operating room efficiency, assess surgical skills, and assist with surgical training and education [3]. Using surgical phase recognition during surgery, one can monitor the progress of the procedure, provide context-aware decision support, detect potential deviations and anomalies, perform objective and data-driven analysis of workflow and compare best practices [4]. However, even for advanced computer-assisted teaching systems [5, 6], the task of identifying the surgical phase from intraoperative video remains challenging due to the diversity of patient anatomy and surgeon styles [7] and the limited availability and quality of video material [8]. In addition, the high degree of similarity between phases and the temporal variations can lead to degraded performance and limited generalization capability of the surgical assist system.

Existing studies mainly focus on modeling high-dimensional visual features or the time sequence information for surgical phase recognition. In terms of visual feature extraction, early studies used manually designed descriptors to extract features, such as intensity and gradient [9], shape, color, and texture-based features [10]. Meanwhile, in time sequence feature modeling, several studies have utilized linear statistical models to capture the temporal structure of surgical videos, including dynamic time warping [11, 12], conditional random fields [13–15], and variants of hidden Markov models (HMMs) [16, 17]. However, since manually designed descriptors are highly time-consuming and rely heavily on manual tuning in processing video frames, they fail to satisfy the needs of fast automated surgical video understanding.

To address these limitations, several deep learning-based methods have been proposed for surgical video understanding, where deep learning methods possess faster surgical phase recognition than manually designed descriptors and do not require manual tuning of filter parameters. For example, Twinanda et al. [18] proposed EndoNet, which employs AlexNet as the backbone for surgical phase recognition. Subsequently, Jin et al. [19] proposed an end-to-end recurrent convolutional network for online cholecystectomy video recognition, realizing that visual representations and sequential dynamics can be jointly optimized in the learning process. Czempiel et al. [20] introduced a multi-stage temporal convolutional network, which consists of multiple temporal convolutional layers for extracting temporal features. The temporal convolutional network has a larger receptive field, which allows the network to obtain longer temporal information. Shi et al. [21] proposed an attention-based spatiotemporal neural network consisting of a spatial model and a temporal model for accurate identification by end-to-end training. In addition, several studies have attempted to improve the surgical phase recognition performance by forming a multi-task learning or multi-modal learning framework. For example, Jin et al. [22] proposed regarding surgical phase classification as a multi-task pattern, where the extracted video features are used for surgical instrument detection and surgical phase recognition, respectively. However, performing surgical phase recognition in a multi-task fashion requires additional labels, which increases the workload of data annotation. Moreover, in surgical practice, numerous video frames with indistinguishable visual characteristics exist, i.e., hard frames, which are assigned different labels. To this end, Yi et al. [23] proposed treating hard frames as mislabeled samples and finding these hard frames in the training set by a data cleaning strategy and then handling the detected hard frames separately by an online hard frame mapper to mitigate the negative effects of hard samples. However, the lack of modeling of long time sequences makes this type of method classify all extremely similar phases all as hard frames, thus making it difficult to further improve their performance. The above methods all use LSTM [24] to capture time information, which retains a finite sequence of memories that cannot span minutes or hours, which is the average duration of surgeries.

With the success of temporal convolutional networks (TCNs) in speech synthesis [25, 26], many researchers have used similar ideas for temporal action segmentation tasks. Compared to RNNs, TCNs better capture the remote dependencies between video frames by relying on large perceptual fields. Later, a multi-stage temporal convolutional network (MS-TCN) [27] was introduced for action segmentation and consists of multiple stages, where each stage outputs an initial prediction that is refined by the next stage. Each stage has a set of dilated temporal convolutions to generate an initial prediction, which is refined by the next stage. Li et al. [28] proposed an improved version of the model based on the MSTCN, called MS-TCN++, which possesses a dual dilated layer that combines both large and small receptive fields to capture both local and global features.

**Fig. 1:**
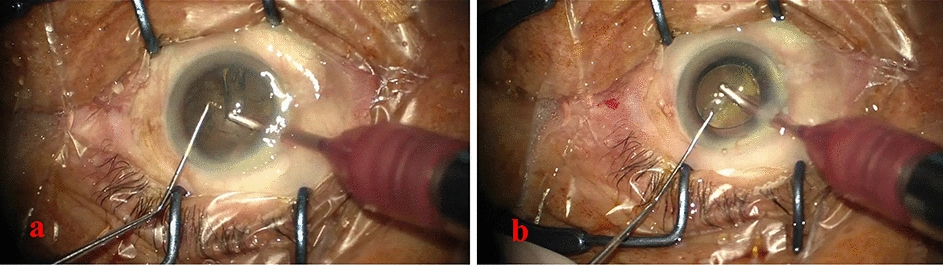
Video frames of different surgical phases with similar appearances. **a** Represents phase 5: irrigation and aspiration, and **b** indicates phase 8: viscous agent removal

The high similarity between the phases of cataract videos lies in the high similarity to the surgical context. When capturing the cataract surgery video, the microscopic camera only focuses on a limited field of view around the human eye, which results in an extremely similar background throughout the video [29]. In addition, this surgical procedure requires delicate operations, causing the differences between each step to be extremely difficult to distinguish. Cataract surgery can be divided into 9 phases [29]: *incision* (P1), *rhexis* (P2), *hydrodissection* (P3), *phacoemulsification* (P4), *irrigation and aspiration* (P5), *viscous agent injection* (P6), *lens implant setting-up* (P7), *viscous agent removal *(P8), and *tonifying and antibiotics* (P9). In these phases, the same surgical instruments may appear in different surgical phases and the variations in crystalline lens appearance are not obvious, as illustrated in Fig. 1, which significantly increases the difficulty of identifying the surgical phases. Another main challenge is the temporal variations of phases across cataract surgery videos. Due to the clinical experience of the surgeons and the condition of the patients, the duration of each video and even each phase varies greatly [30]. Furthermore, the imbalance of time span between surgical video phases makes it harder to recognize surgical phases with a shorter time span.

To address the aforementioned limitations, we introduce a global–local multi-stage temporal convolutional network (GL-MSTCN) for challenging surgical phase recognition by extracting fine-grained features of video frames and varying lengths of time span features, respectively. This paper makes three contributions:

- We propose a triple-stream network (TS-Net), pupil stream, instrument stream, and video frame stream, to increase the distance between similar surgical phases by extracting global–local fine-grained features. The pupil stream and surgical instrument stream can extract fine-grained features in the pupil and surgical instrument patches acquired by a YOLOv3 [31] detector, thus aiding the video frame stream in better distinguishing between extremely similar surgical phases.
- To improve the robustness of the model in identifying surgical phases with various durations of surgical videos and unbalanced time spans between different phases, we propose a residual multi-stage temporal convolutional network to exploit the long-range temporal dependence of different surgical phases. Furthermore, we adopt a dual dilated layer in the proposed residual multi-stage temporal convolutional network to capture the local features of transition frames of adjacent surgical phases and the global features of each phase to improve the surgical phase recognition performance.
- The proposed method is validated on a cataract surgery video dataset including a total of 32 videos with different surgeons and different time durations, and the quantitative results demonstrate the state-of-the-art performance of the proposed method.

The remaining of the paper is organized as follows: “Results” section shows the statistical and quantitative results of our proposed method. In “Discussion” and “Conclusion” sections, detailed discussions and conclusions are presented. The proposed method is described in “Methodology” section, including the experimental settings and evaluation measures.

## Results

In this section, we perform hold-out validations [32] and ablation studies to verify the feasibility of the proposed method. All experiments were repeated 5 times with random initialization to ensure reproducibility of the results.

### Evaluation metrics

To better quantify the proposed method, we follow [22] and choose the *Accuracy*, *Precision*, *Recall* and *Jaccard* metrics to evaluate the recognition performance, i.e.,

1 $$\begin{aligned} \text{Precision} &=\frac{1}{C}\sum _{c=0}^C \frac{\text{TP}_c}{\text{TP}_c+ {\text{FP}}_c},\\ \text{Recall} &=\frac{1}{C}\sum _{c=0}^C \frac{\text{TP}_c}{\text{TP}_c+{\text{FN}}_c},\\ \text{Jaccard} &=\frac{1}{C}\sum _{c=0}^C \frac{\text{TP}_c}{\text{TP}_c+\text{FN}_c+{\text{FP}}_c},\\ {\text{Accuracy}} &=\frac{1}{C} \frac{\sum _{c=0}^C \text{TP}_c}{\sum _{c=0}^C(\text{TP}_c+{\text{TN}}_c+{\text{FN}}_c+{\text{FP}}_c)},  \end{aligned}$$

where $\text{TP}_c$, $\text{TN}_c$, $\text{FP}_c$,and $\text{FN}_c$ represent the true-positive, true-negative, false-positive, and false-negative samples of surgical phase *c* and *C* is the total number of phases.

### Comparison with state-of-the-art methods

To quantify the performance of the proposed GL-MSTCN, we compared it with several state-of-the-art methods, including ResNet50 [33], OHFM [23], SV-RCNet [22], STANet [21], and TeCNO [20], using holdout validation. The quantification results are shown in Tables 1 and 2. By observing the comparison results in Tables 1 and 2, the proposed GL-MSTCN achieves state-of-the-art performance on the CSvideo and Cataract101 datasets, respectively.

**Table 1:** Classification performance of different methods on **CSVideo**

Methods	Accuracy	Precision	Recall	Jaccard
ResNet50 [33]	0.905	0.899	0.900	0.811
OHFM [23]	0.923	0.928	0.914	0.851
SV-RCNet [22]	0.941	0.937	0.942	0.882
TeCNO [20]	0.930	0.931	0.931	0.866
STANet [21]	0.948	0.941	0.941	0.887
GL-MSTCN	0.958	0.951	0.953	0.907

**Table 2:** Classification performance of different methods on **Cataract101**

Methods	Accuracy	Precision	Recall	Jaccard
ResNet50 [33]	0.864	0.828	0.823	0.710
Qi et al. [34]	0.881	-	-	-
OHFM [23]	0.920	0.892	0.903	0.816
SV-RCNet [22]	0.934	0.913	0.922	0.848
TeCNO [20]	0.936	0.917	0.915	0.847
STANet [21]	0.953	0.934	0.935	0.879
GL-MSTCN	0.965	0.949	0.952	0.908

Among these comparison methods, SV-RCNet integrates ResNet50 [33] and LSTM to jointly learn spatial and temporal features. Due to the limitation of computing resources, SV-RCNet can only capture time information within a small video segment. We introduce the multi-stage temporal convolutional networks in our model, which can capture the long-range temporal dependence between all frames in an entire cataract surgery video, as evidenced by the 1.7% and 2.5% improvement in accuracy and Jaccard score of the proposed model over SV-RCNet, respectively. In STANet, an attention-based spatial–temporal neural network architecture was proposed for better surgical phase recognition. However, as with SV-RCNet, STANet can only capture the temporal features of short-time span video segments, resulting in a great limitation of the performance, as STANet cannot capture sufficient long-time span temporal information. By observing the quantitative comparison in Table 1, the Accuracy, Precision, Recall, and Jaccard score of the proposed method are 1.0%, 1.0%, 1.2%, and 2.0% higher than those of STANet. Unlike SV-RCNet and STANet, OHFM uses ResNet50 to extract semantic features and employs a three-step strategy to mitigate the negative impact of hard samples on classification. However, the OHFM only roughly treats misidentified frames as hard frames, resulting in its inability to distinguish between different phases across numerous similar frames. This also leads to the worst performance of the OHFM in classifying cataract surgery phases. To obtain more long-range temporal dependencies, TeCNO uses an online feature learning method based on a CNN and TCN to explore long-range temporal relationships in precomputed spatial features. However, TeCNO only obtains long-range temporal dependencies by simply using a TCN while neglecting to explore the local fine-grained information between similar frames. Unlike TeCNO, we introduce a dual dilated temporal convolutional layer in the multi-stage temporal network to obtain global and local temporal information of video frames. By observing the quantitative comparisons in Table 1, the GL-MSTCN outperforms TeCNO by 2.8%, 2.0%, 2.2%, and 4.1% in terms of Accuracy, Precision, Recall, and Jaccard, respectively.

Table 2 reports the surgical phase recognition results of our GL-MSTC and other deep learning methods on the public Cataract101 dataset. In Table 2, we additionally added Qi’s method as a comparison method, which relies on the extracted edge information and the spatial information in the original image and is the first surgical phase recognition method applied to the Cataract101 dataset. The GL-MSTCN outperforms Qi’s method in terms of Accuracy by 7%, which demonstrates that the performance of surgical phase recognition can be improved by aggregating temporal information into spatial features. Moreover, the GL-MSTCN achieves the best performance among all methods (95.8% of accuracy, and 90.7% of Jaccard index). It achieved, more than 1% higher accuracy and Jaccard index, compared with other surgical stage recognition networks in terms of accuracy and Jaccard.

Overall, our proposed method exhibits better performance in identifying cataract surgery phases. Embedding temporal convolutional layers in our method enables the proposed method to model the features of surgical phases of different durations, which further enhances its ability to discriminate between excessive phases.

### Ablation study

To verify the effectiveness of the proposed TS-Net, we conduct ablation studies to quantify the performance of each stream in the proposed TS-Net. First, we verify the effectiveness of the local feature extraction performance of the instrument stream (IS) and pupil stream (PS) by removing IS (denoted TS-Net w/o IS) and PS (denoted TS-Net w/o PS) from TS-Net. Next, we verify the effectiveness of $S_1$ by embedding it behind TS-Net (denoted TS-Net w/$S_1$) and the effectiveness of $S_2$ by plugging it behind TS-Net w/$S_1$ [denoted TS-Net w/$(S_1,S_2)$]. The evaluation results are demonstrated in Table 3, where ResNet50 represents the backbone of the proposed TS-Net. The comparisons in Table 3 show that the performance of the backbone network is improved with the integration of surgical instruments and pupil streams. Moreover, the surgical phase recognition performance is further improved after $S_1$ and $S_2$ are plugged into TS-Net. Therefore, we can conclude that the proposed triple-stream network can better focus on extracting global information of video frames and local fine-grained information of surgical instruments and the pupil, which also helps the GL-MSTCN obtain robust temporal information.

**Table 3:** Ablation study of key components of the proposed method

Methods	Accuracy	Precision	Recall	Jaccard
ResNet50 [33]	0.905	0.899	0.900	0.811
TS-Net w/o IS	0.919	0.917	0.908	0.844
TS-Net w/o PS	0.916	0.904	0.909	0.833
TS-Net	0.928	0.910	0.910	0.837
TS-Net w/$S_1$	0.941	0.931	0.937	0.882
TS-Net w/($S_1,S_2$)	0.958	0.951	0.953	0.907

### Typical case study

**Fig. 2:**
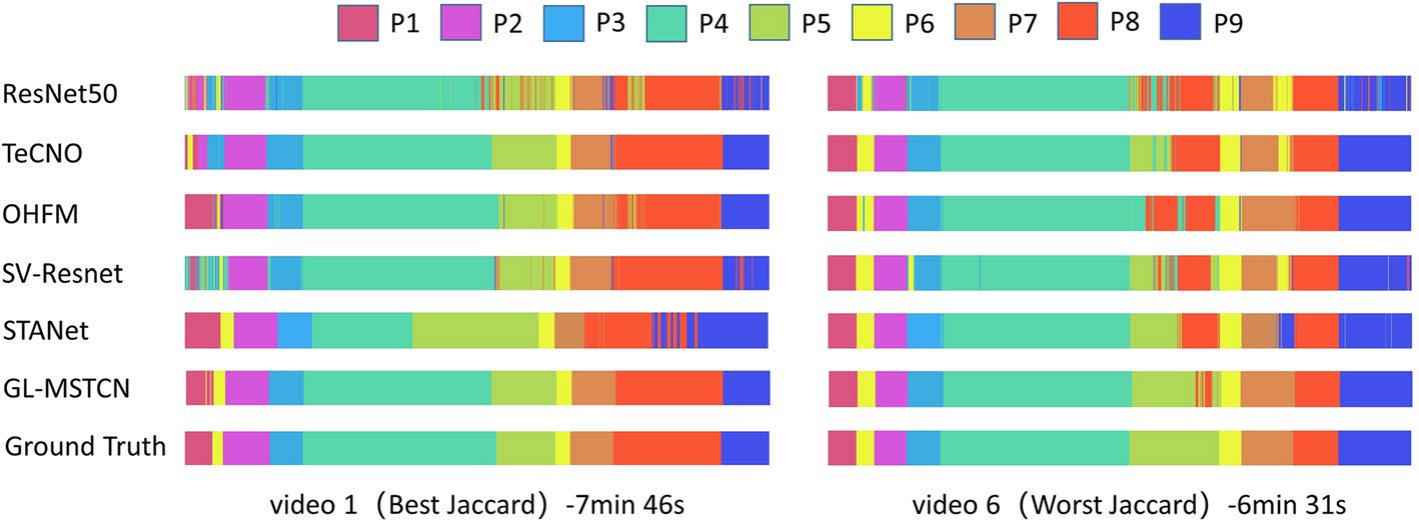
Color-coded ribbon illustration of nine phases (P1–P9) during two complete surgical videos, whose horizontal axis represents the time progression. In each case, from top to bottom are the results from our four comparison methods, the GL-MSTCN, and the ground truth

Figure 2 illustrates the classification results of complete surgical videos in the color-coded ribbon. In test video 1 shown in Fig. 2, the proposed GL-MSTCN obtained the best Jaccard index, and the proposed method can accurately identify most phase transitions with a deviation of fewer than 15 s. The proposed GL-MSTCN can reduce the deviation by 17 seconds, 13 seconds, and 24 seconds compared with TeCNO, OHFM, and SV-RCNet, respectively. Since the residual multi-stage temporal convolutional network has a larger receptive field and full temporal resolution, it is more effective for long-time span phase identification, as demonstrated by Fig. 2, and has the longest time span identification for P6. Moreover, we introduce a dual dilated layer into the residual multi-stage temporal network, which improves the accuracy of transitions from P6 to P2, P4 to P5, and P7 to P8 and provides smoother and more accurate estimates in transition frames. This is important for computer- and robot-assisted surgeries to prepare for the next stage, such as automatically adjusting configuration parameters in advance. We also demonstrate the phase classification results for the video (video 6) with the worst Jaccard score among all tested videos. The visualization of surgical phase recognition for video 6 in Fig. 2 shows that P5 is usually misclassified as P8. The result is mainly attributed to the high degree of similarity between P5 and P8, with the same tools being used and the backgrounds being similar, as shown in Fig. 1. Compared with other methods, our proposed method achieved the best performance for the classification of P5 and P8 on video 6, which demonstrates that our model has better performance in the discrimination of similar frames.

## Discussion

Automatic surgical phase recognition plays an essential role in modern smart operating rooms; however, the high similarity between the phases and temporal variations of cataract videos pose challenges for surgical video phase recognition. In this paper, we propose a global–local multi-stage temporal convolutional network (GL-MSTCN) for surgical phase recognition, which improves recognition performance by fusing captured local fine-grained information with global information over large time spans. Most existing methods widely use LSTM networks and 3D CNNs to analyze the temporal space of surgical videos, resulting in the inability to observe the long-range temporal dependency, while the proposed GL-MSTCN utilizes a multi-stage temporal convolutional network to capture complex multi-scale temporal patterns. The experimental results demonstrate that the proposed network can improve the phase recognition performance of cataract surgery videos.

**Fig. 3:**
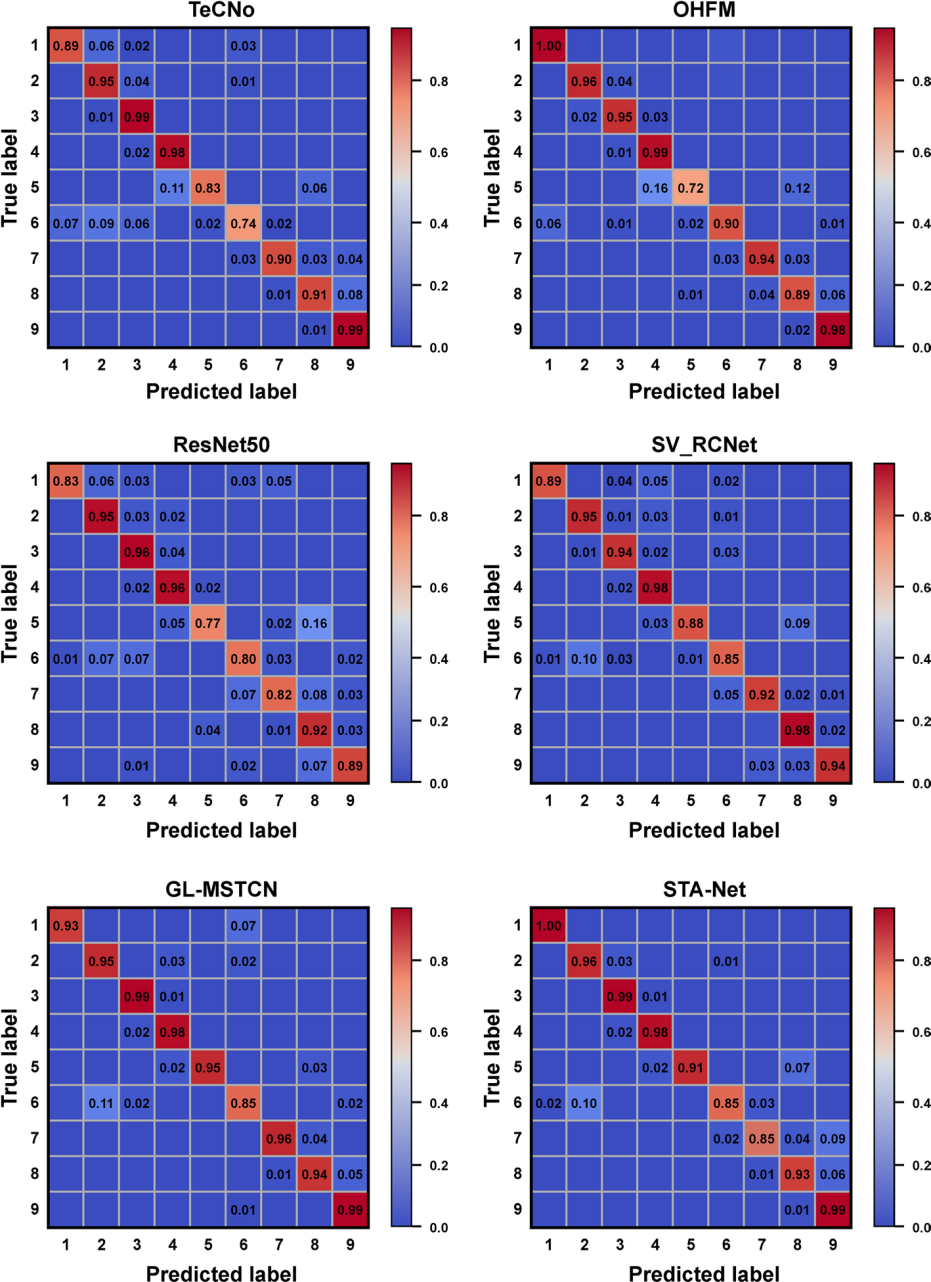
Confusion matrices of different methods in surgical phase recognition

Due to the limited camera field of view, the relatively fixed location of the crystalline lens in the camera’s limited field of view and the inconspicuous variation in the appearance of surgical instruments, frames of different phases with similar spatial–visual characteristics are likely to be incorrectly predicted as the same surgical phase. Previous work either used conventional CNNs to directly identify cataract surgery stages [30] or used CNNs to first identify surgical instruments in video frames and then perform temporal regularization using LSTM [24]. However, these efforts are not ideal for identifying similar frames because the same surgical instruments appear in different surgical stages during cataract surgery and changes in the environment occur during surgery. In contrast, the feature extraction backbone TS-Net in our GL-MSTCN uses the fusion of local and global features to enable the network to exploit the complementary local and temporal information to produce more discriminative visual features. Figure 3 illustrates the confusion matrices of the proposed method and the comparison methods in recognizing the surgical phases. Since phase 5 and phase 8 share extremely similar visual features (as shown in Fig. 1), ResNet50 incorrectly identifies phase 5 as phase 8, as demonstrated in Fig. 3. The confusion matrix of the GL-MSTCN demonstrates that TS-Net can strengthen the recognition performance of similar visual frames by extracting local fine-grained features through surgical instruments and pupil streams. However, although TS-Net improves the fine-grained feature extraction capability, there is room for considerable improvement for the recognition of certain phases (e.g., phases 5 to 8), which can also be observed in the OHFM, TeCNO, and SV-RCNet, respectively.

Several previous works (e.g., the OHFM and SV-RCNet) used LSTM or other RNNs networks to capture the temporal features between different phases, but they retained the memory of a limited sequence that could not span minutes or hours, which is the average duration of the surgeries. Since the time span between phase 5 and phase 8 is relatively long (i.e., usually 1–2 min), conventional LSTM fails to memorize temporal information of such a long time span. With the assistance of a global–local multi-stage temporal convolutional network (GL-MSTCN), the proposed method can capture ultralong-time span temporal information via the exponentially increased dilated temporal convolutional layer. The proposed GL-MSTCN enhances the discrimination of different phases by capturing temporal information, thus improving the recognition performance, as demonstrated by the confusion matrix of the GL-MSTCN in Fig. 3.

Although the proposed method shows promising applications, there are still a few limitations that need to be mentioned. (a) The cataract surgery video data included in this paper were from a single hospital, resulting in less data diversity. In future work, we intend to include a wider range of data from different surgeons and different hospitals. (b) The performance is not satisfactory for the recognition of similar frames without any surgical instruments and with less obvious crystalline lens changes (e.g., interstitial frames in the stage of changing instruments). Due to the current limitations described above, data cleaning of frames without surgical instruments will be required in subsequent studies. Furthermore, to enhance the generalizability of the method, it is necessary to include a wider range of diverse databases to perform a comprehensive validation.

## Conclusion

In this paper, we propose a global–local multi-stage temporal convolutional network to address the performance limitations due to the high similarity of different phases in cataract surgery. The proposed TS-Net is designed to extract fine-grained features of video frames, which allows better exploration of more representative spatial details between different phases. At the same time, the proposed GL-MSTCN uses temporal dilated convolutional layers to obtain full temporal resolution by increasing the temporal receptive fields. Specifically, we introduce a dual dilated temporal convolutional layer to explore the local semantic information between adjacent phases and the global long-range temporal dependencies of each phase. Extensive experimental results show that the proposed GL-MSTCN can improve phase recognition in cataract surgery and achieve state-of-the-art performance. This approach has great potential to be introduced into AI systems for surgical skills assessment and computer-assisted surgery (CAS) systems to assist the surgeons in avoiding technical errors and provide real-time information for better decision-making. Overall, the strategy of our proposed method allows us to use existing object detection methods to preextract fine-grained features to assist the model in better video phase identification. In future work, we would like to focus on collecting more cataract surgery videos from different medical centers to validate and strengthen the generalization capability of our proposed method. Furthermore, extending the proposed method to other types of surgical video analysis tasks is also one of the future works.

## Methodology

**Fig. 4:**
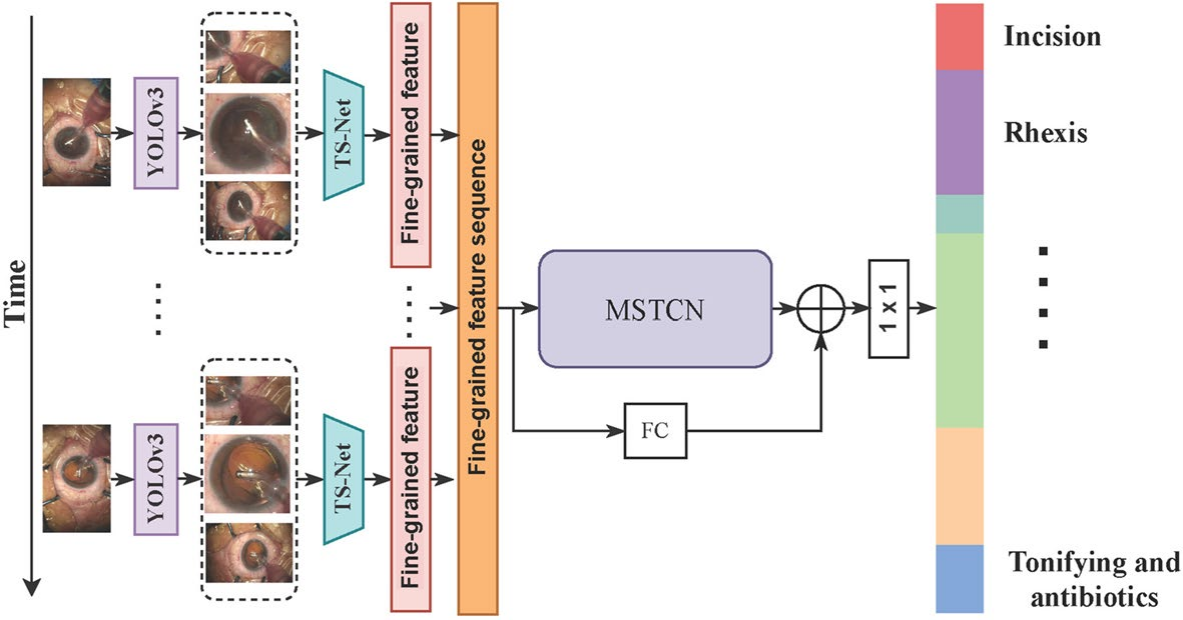
Flowchart of our proposed method

**Fig. 5:**
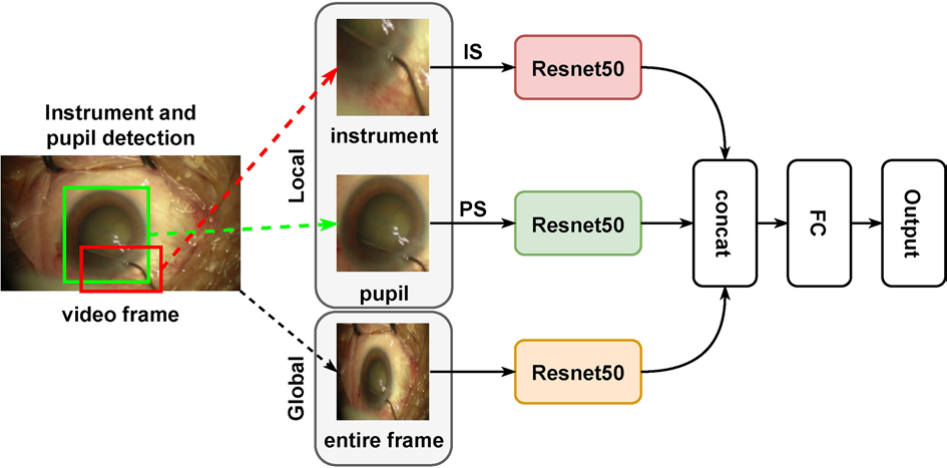
Schematic diagram of the proposed TS-Net. The surgical instruments and the pupil in the video frame are detected and isolated using YOLOv3 [31]. IS and PS indicate the instrument stream and pupil stream, respectively

In this paper, we propose a network consisting of a triple-stream network (TS-Net) and a multi-stage dilated temporal convolutional network (MSTCN [35]). The former is used to extract global–local features from cataract surgery videos, and the latter is used to capture long-range temporal dependencies of cataract surgery videos. A flowchart of the proposed GL-MSTCN is illustrated in Fig. 4. First, we use a fine-tuned YOLOv3 [31] to extract pupil and surgical instrument patches in video frames. Second, we input the extracted patches and the video frames into a triple-stream network (TS-Net) (as illustrated in Fig. 5) to extract the global and local semantic information of the similar-looking video frames. For each frame, the TS network generates a fine-grained feature vector. Then, the fine-grained feature vectors of all frames are combined into a vector sequence (denoted $\mathbf {v}_{\text{s}}$) representing the fine-grained features of the entire video. Next, the vector sequence is input into the MSTCN [35] to capture longer sequence information through progressively increased receptive fields. Subsequently, we pass the vector sequence $\mathbf {v}_{\text{s}}$ through a fully connected layer and then perform residual learning [33] with the features extracted by the MSTCN [35] to obtain the output of the proposed GL-MSTCN. Finally, the GL-MSTCN extracted features are fed to a classifier for cataract video phase classification.

### Architecture of TS-Net

Since cataract surgery videos have high similarity to the surgical context and the same surgical instruments may appear in different surgical phases, the precise recognition of surgical phases is extremely challenging. To address these limitations, we propose a triple-stream network (i.e., TS-Net) to exploit discriminative fine-grained features. The detailed architecture of TS-Net is illustrated in Fig. 5.

First, we use a fine-tuned YOLOv3 [31] to obtain the pupil and surgical instrument patches with fine-grained features in a single video frame. For the training of YOLOv3, we input the video frames with the bounding box labels of the pupil and surgical instruments to fine-tune the model. Then, we use the obtained pupils and surgical instrument patches along with video frames as the inputs to the pupil feature extractor stream, the instrument feature extractor stream, and the video frame feature extractor stream. The ResNet50 [33] model serves as the feature extraction backbone in each stream. Second, the output features from the fully connected layers of these three streams are concatenated as a fine-grained feature representation of the whole frame. Finally, the concatenated features are fed into a classifier (i.e., a fully connected layer) to generate preliminary predictions of cataract surgery phases. With the above steps, our proposed TS-Net is capable of simultaneously exploiting the global information of video frames as well as the local fine-grained information contained in pupil and surgical instrument patches. The proposed TS-Net can improve the recognition of hard frames by learning the pupils and surgical instrument features that appear in different surgical phases and by selectively classifying them based on pupils or surgical instruments when the phase cannot be correctly predicted from a single video frame.

### Architecture of the MSTCN

**Fig. 6:**
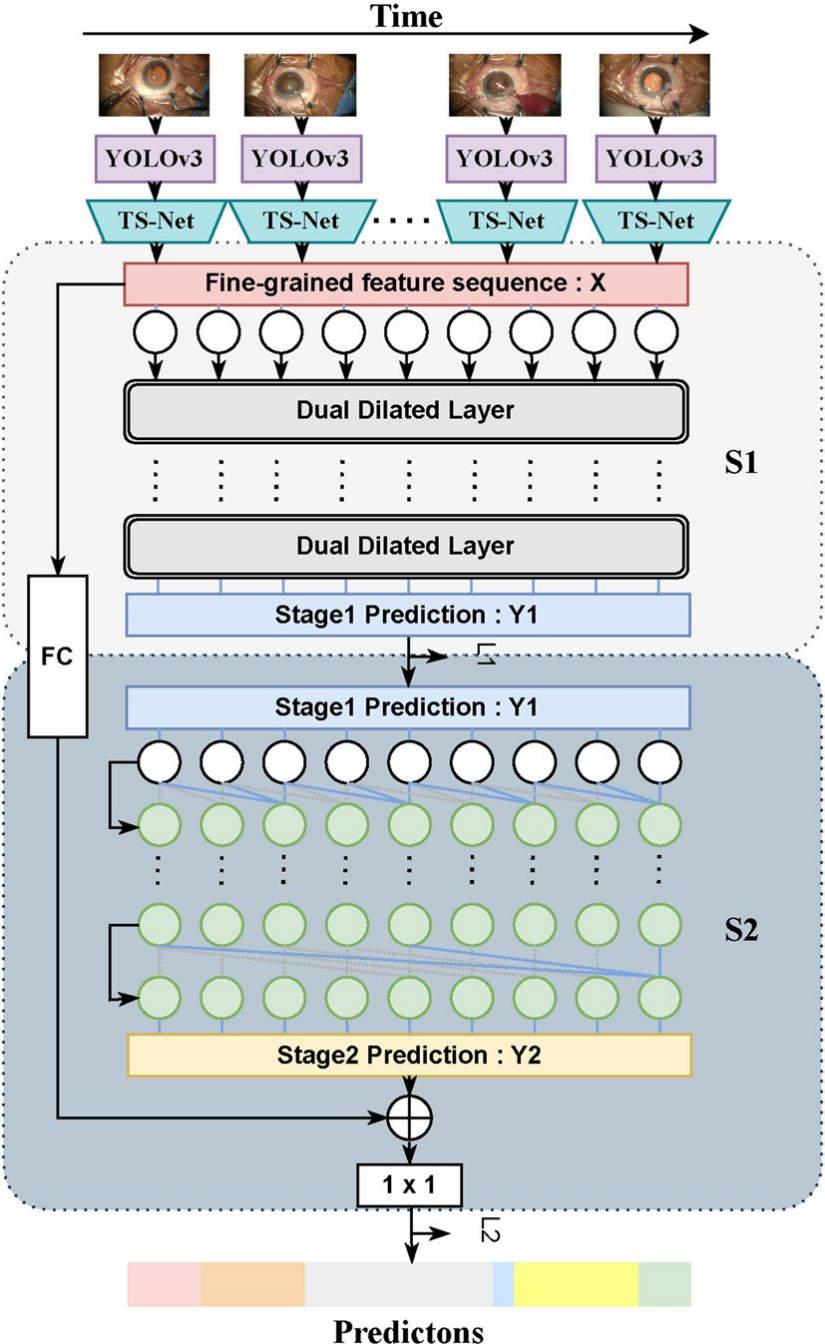
Overview diagram of the proposed GL-MSTCN. The proposed GL-MSTCN contains a global–local fine-grained features extraction network (TS-Net) and a multi-stage dilated temporal convolutional network (MSTCN). The MSTCN consists of $S_1$ and $S_2$, where $S_1$ and $S_2$ are composed of parallel dual dilated convolutional layers and single dilated convolutional layers, respectively. $L_1$ and $L_2$ denote the loss functions of $S_1$ and $S_2$, respectively. FC is a fully connected layer

The existing study has demonstrated that stacking multiple predictors can significantly improve the performance of the model [27]. Inspired by [35], we propose a residual multi-stage temporal convolutional network (MSTCN) to predict temporal phases. The MSTCN consists of two stages: the first stage (denoted $S_1$) is composed of *N* dual dilated temporal convolutional layers, and the second stage (denoted $S_2$) is composed of *N* single dilated temporal convolutional layers, as shown in Fig. 6. To capture the entire time information of the video, we set *N* to 9. Specifically, the input of $S_1$ is the fine-grained semantic features of each frame extracted by the proposed TS-Net, $X=(x_1,x_2,\ldots ,x_t),t\in [1,T]$, where *T* is the total number of frames. We then denote the output feature of $S_1$ $Y_1=\Gamma _{S1}(x_1,x_2,\ldots ,x_t),t\in [1,T]$, where $\Gamma _{S1}(\cdot )$ denotes the dual dilated temporal convolutional layer. Subsequently, we use $Y_1$ as the input to $S_2$ to obtain the output $Y_2$, which is then concatenated with *X* and fed into a $1\times 1$ convolutional layer for dimensionality reduction. Finally, $Y_2$ is fed into the classifier for the final surgery phase prediction.

In addition, we replace non-causal convolution in the MSTCN [35] with causal convolution in the dilated temporal convolution layer. Unlike the non-causal convolutional, in which prediction $\hat{y}_t$ for time step *t* depends on *n* past and *n* future frames, the prediction $\hat{y}_t$ of causal convolution does not depend on any *n* future frames, but depends only on the current frame and previous frames, i.e., $\hat{y}_t(x_{t-n},\cdots ,x_t)$. This allows the GL-MSTCN to be deployed in an online computer-assisted surgery (CAS) system.

**Fig. 7:**
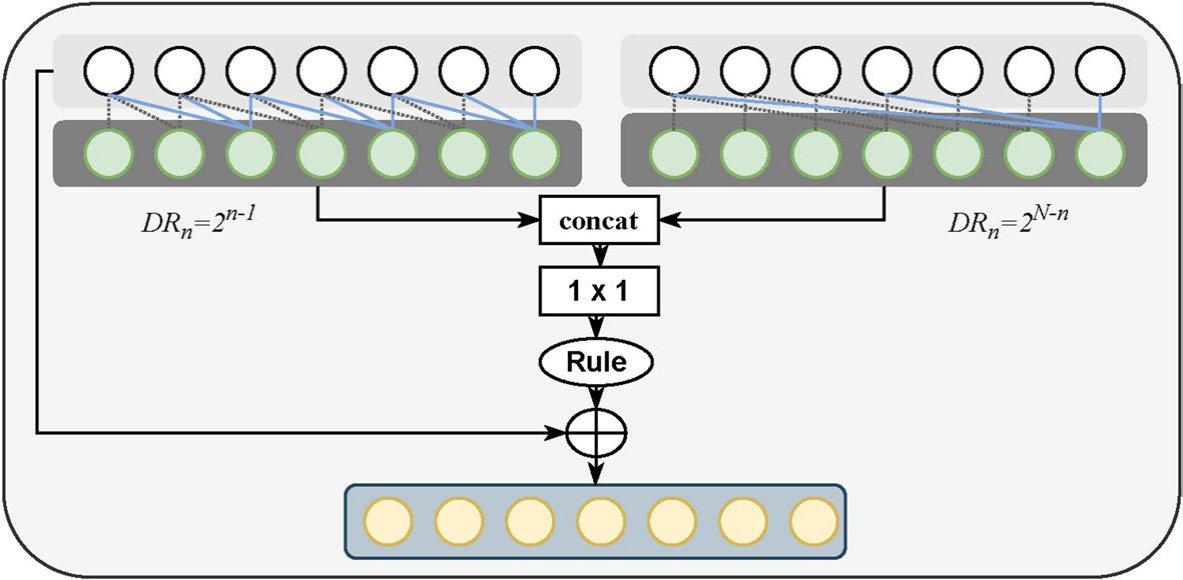
Schematic diagram of $n{\text{th}} (n\in [1,N])$ dual dilated temporal convolutional layer. The dual dilated layers use two different sets of dilation rates, one increasing with the number of layers and the other the opposite

In the first stage, the introduced dual dilated temporal convolutional module follows the design of MS-TCN++ [28], as shown in Fig. 7. The dual dilated temporal convolutional module contains two convolutional layers with different dilation rates. The dilation rate of the first layer increases exponentially as the number of layers increases $\text{DR}_n=2^{n-1}$, where $\text{DR}_n$ indicates the dilation rate of the $n{\text{th}}$ layer. The other dilated temporal convolutional layer show the opposite trend of the first layer. That is, the dilation rate decreases exponentially as the number of layers increases: $\text{DR}_n=2^{N-n}$. Each layer applies a dilated convolution with ReLU activation to the output of the previous layer while using residual connections to facilitate gradient flow. Mathematically, the set of operations for each layer can be represented as follows:

2 $$Q_{l,d_1}=W_{1,l}*D_{l-1}+b_{1,l}, $$

3 $$Q_{l,d_2}=W_{2,l}*D_{l-1}+b_{2,l}, $$

4 $$Q_l={\text{ReLU}}(\text{LayerNorm}([Q_{l,d_1},Q_{l,d_2}])), $$

5 $$D_l=D_{l-1}+W_{3,l}*Q_l+b_{3,l}, $$

where $Q_{l,d_1}$ and $Q_{l,d_2}$ are the output of the first and the second dilated temporal convolutional layer with weights ($W_{1,l}$ and $W_{2,l}$) and biases ($b_{1,l}$ and $b_{2,l}$), respectively. $Q_l$ indicates the concatenation of $Q_{l,d_1}$ and $Q_{l,d_2}$ followed by a $1\times 1$ convolutional layer, normalization (LayerNorm) and ReLU activation. D$_l$ is the output of the $l{\text{th}}$ dual dilated temporal convolutional layer, where $W_{3,l}$ is the weight of the $1\times 1$ convolutional layer with bias $b_{3,l}$ in Eq. 4, $*$ denotes a convolutional operator.

The second stage consists of *N* dilated temporal convolution layers, where each layer is followed by layer normalization and ReLU activation. Similarly, the dilation rate of the intermediate temporal convolution layer increases exponentially, i.e., the dilation rate of the $n{\text{th}}$ layer is $\text{DR}_n=2^{n-1}$.

### Loss function

The identification of surgical phases has an unbalanced pattern due to the large variation in the number of video frames in each phase of cataract surgery videos. Therefore, we employ the weighted cross-entropy loss to train, TS-Net to address the imbalance in the convergence speed of the loss function of the deep learning model by assigning different weights to different surgery phases. Mathematically,

6 $$\mathcal {L}_{\text{TS-Net}}=-\frac{1}{N} \sum _{i=0}^N \sum _{c=0}^C \omega _c y_{i,c} \cdot \log \hat{y}_{i,c}, $$

where $y_{i,c}$ and $\hat{y}_{i,c}$ indicate the ground truth and predicted probability of the $i{\text{th}}$ frame belonging to class *c*, respectively. *N* denotes the number of all frames, while *C* denotes the number of classes, i.e., the number of surgical video phases. The class weight of the $c{\text{th}}$ class $\omega _c$ is obtained by using median frequency balancing [36]. To train the GL-MSTCN, we also employ the weighted cross-entropy loss as the cost function:

7 $$\begin{aligned}\mathcal {L}_{\text{MSTCN}}&=\frac{1}{M}\sum _{m=1}^M L_{m}\\&=-\frac{1}{M}\frac{1}{N}\sum _{m=1}^M \sum _{i=0}^N \sum _{c=0}^C \omega _c^{(m)} y_{i,c}^{(m)} \cdot \log \hat{y}_{i,c}^{(m)}, \end{aligned}$$

where $L_{m}$ is the loss of the $m{\text{th}}$ ($m\in \{1,M\}$) stage, *M* denotes the number of stages of the GL-MSTCN, and $y_{i,c}^{(m)}$ and $\hat{y}_{i,c}^{(m)}$ indicate the ground truth and predicted probability of the $i{\text{th}}$ frame belonging to class *c* in the $m{\text{th}}$ stage, respectively. Similarly, the class weight of the $c{\text{th}}$ class in the $m{\text{th}}$ stage $\omega _c^{(m)}$ is also obtained by using median frequency balancing [36].

### Dataset

**Fig. 8:**
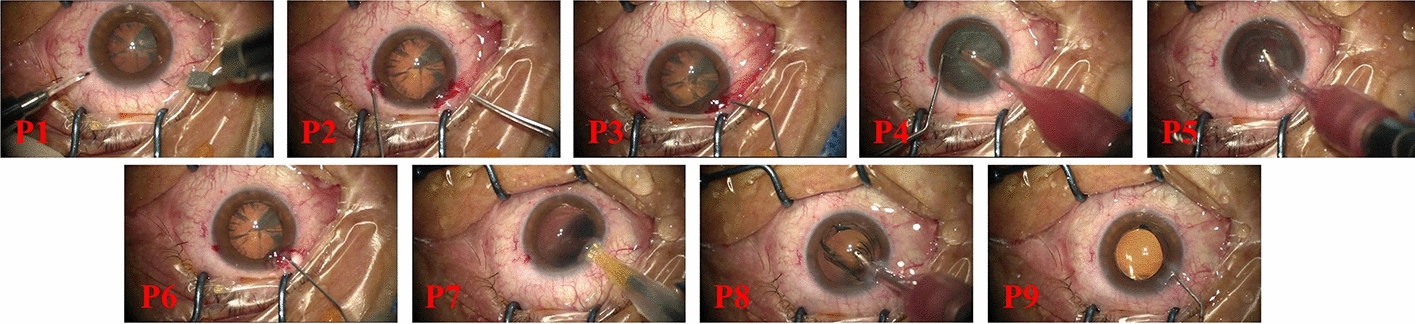
Sample of nine phases in CSVideo dataset. Incision (P1), rhexis (P2), hydrodissection (P3), phacoemulsification (P4), irrigation and aspiration, viscous agent injection (P6), lens implant setting-up (P7), viscous agent removal (P8), and tonifying and antibiotics (P9)

**CSVideo**. An in-house **cataract surgery video** (**CSVideo**) dataset acquired from a local hospital was used to train, validate and test the surgical phase recognition model. The CSVideo dataset includes 32 videos of cataract surgeries from different surgeons. Each video is annotated by an experienced ophthalmic surgeon (with more than 10 years of clinical experience) into nine surgical phases based on clinical experience and previous studies [29], as shown in Fig. 8. All the videos were captured by an ophthalmic operating a microscope at a frame rate of 60 fps and $1920\times 1080$ pixels. The average duration of all videos is 6 min, with a maximum of 10 min and a minimum of 4 min. We randomly selected 22, 4, and 6 videos from 32 videos as the training set, validation set, and test set, respectively. To reduce GPU consumption, we downsampled the frame rate of the video to 20 fps and resized each frame to $720\times 480$ pixels.

**Cataract101**. To verify the reproducibility and generalizability of our method, we introduced a large public surgical video dataset, the Cataract-101 dataset  [37]. The dataset contains 101 videos of surgeries performed by four different surgeons (two experienced senior surgeons and two less experienced assistant surgeons). It is annotated with the ground truth of ten quasi-standardized operation phases typically performed for such operations (without serious complications). Following the splitting strategy in [34], We randomly selected the 73 and 28 videos from 101 videos as the training set and test set, respectively. All videos have a frame rate of 25 fps and a resolution of $720\times 540$ pixels. The average length of all videos is 8 min, with a maximum of 17 min and a minimum of 4 min. In order to reduce GPU consumption, we downsampled the frame rate of the video to 1 fps.

### Experimental setup

The proposed method was implemented on the PyTorch platform in Ubuntu 16.04 LTS with a single NVIDIA GPU (GeForce GTX Titan XP). The experimental configurations of TS-Net and the GL-MSTCN are shown in Table 4. Due to the limitation of GPU computing resources, we set the batch size to 16 when training TS-Net and we use adaptive moment estimation (Adam) with a decay of 0.0005 as the optimizer. We use poly learning rate decay strategy with an initial learning rate of 0.0005 and power of 0.9: $\text{lr}= \text {init}_{\text {lr}} \times \left( 1-\text{ iters}/\text{total-iters}\right) ^{\text{ power}}$. Since we used pre-trained parameters when fine-tuning TS-Net, the maximum epoch was set to 50. For the training of the GL-MSTCN, we set the maximum epoch, the initial learning rate, and the batch size to 200, 0.0002, and 1, respectively. All experiments were repeated 5 times with random initialization to ensure the reproducibility of the results. During the fine-tuning of the TS-Net, all frames were randomly cropped to $480\times 480$ pixels and subsequently resized to $224\times 224$ pixels. We performed data augmentation using random horizontal and vertical flips with a probability of 0.5 and random rotations with a probability of 0.5 at a random angle within $[-30^{\circ },30^{\circ }]$.

**Table 4:** Experimental configurations

Methods	Lr	Batch size	Optimizer	Epoch
TS-Net	0.0005	16	Adam	50
GL-MSTCN	0.0002	1	Adam	200

## Data Availability

Since the data used in this study include data collected in a clinical trial with patients, the CSVideo dataset is not publicly available.

## Abbreviations

TCN: Temporal convolutional network
MSTCN: Multi-stage dilated temporal convolutional network
LSTM: Long short-term memory
HMM: Hidden Markov models
3D CNN: 3-Dimensional convolutional neural network
CNN: Convolutional neural network
RNN: Recurrent neural network
